# Microbially produced bile acids are associated with increased IgG autoantibodies and poorer mental wellbeing in fibromyalgia

**DOI:** 10.1038/s41598-026-40781-3

**Published:** 2026-02-24

**Authors:** Jenny E. Jakobsson, Henrik Carlsson, Ida Erngren, Joana Menezes, Emerson Krock, Matthew A. Hunt, Jeanette Tour Sohlin, Asma Al-Grety, Katalin Sandor, Eva Kosek, Camilla I. Svensson, Kim Kultima

**Affiliations:** 1 Department of Medical Sciences, Uppsala University, Uppsala, Sweden; 2 Department of Surgical Sciences, Uppsala University, Uppsala, Sweden; 3 Department of Physiology and Pharmacology, Center for Molecular Medicine, Karolinska Institutet, Stockholm, Sweden; 4 Department of Clinical Neuroscience, Karolinska Institutet, Stockholm, Sweden; 5 Department of Medical Sciences, Clinical Chemistry, Uppsala University, Akademiska Sjukhuset Entrance 61 3rd Floor, 751 85 Uppsala, Sweden; 6 Present Address: Faculty of Dental Medicine and Oral Health Sciences, Alan Edwards Centre for Research on Pain, McGill University, Montreal, Canada

**Keywords:** Bile acid, Short-chain fatty acid, Fibromyalgia, Autoimmunity, Metabolomics, Autoimmunity, Neuroimmunology, Gastrointestinal diseases, Rheumatic diseases, Neuroscience, Psychology, Rheumatology

## Abstract

Fibromyalgia (FM) is a disease primarily associated with chronic widespread pain, but other common symptoms are anxiety and depression. We previously proposed that autoimmunity contributes to FM based on findings of increased immunoglobulin G binding to satellite glial cells (anti-SGC IgG) in FM subjects compared to healthy controls (HC). Emerging research suggests that an altered gut microbiota composition is connected to psychological symptoms in FM. Gut microbiota can produce or alter bile acids (BAs) and short-chain fatty acids (SCFAs), which have important immune and inflammatory functions. Here, we investigate alterations in BA and SCFA concentrations in FM subjects compared to HC and potential associations with FM symptoms and anti-SGC IgG levels. Bile acids and SCFAs were quantified using liquid chromatography coupled with high-resolution mass spectrometry and anti-SGC IgG levels were assessed with immunocytochemistry. The correlations between FM symptoms, anti-SGC IgG levels, and serum concentrations of 24 BAs and 11 SCFAs in 35 FM subjects and 32 matched HC were examined. Fibromyalgia subjects had significantly higher levels of non-conjugated microbially produced (secondary) BAs compared to HC. Additionally, total BA levels were significantly elevated in FM subjects with high, compared to those with low, anti-SGC IgG levels. Concentrations of specific BAs were associated with increased disease severity and poorer mental well-being. These results revealed increased levels of non-conjugated secondary BAs in FM subjects compared to HC. The strong association between BAs, anti-SGC IgG levels, and mental well-being may help elucidate the importance of BAs in the psychological symptoms of FM.

**Supplementary Information:**

The online version contains supplementary material available at 10.1038/s41598-026-40781-3.

## Introduction

Fibromyalgia (FM) ranks among the primary causes of chronic widespread pain^1^, affecting approximately 2–5% of people worldwide, with a female preponderance^2,3^. Fibromyalgia and irritable bowel syndrome (IBS) are examples of frequently co-existing nociplastic pain conditions that are typically associated with symptoms such as disturbed sleep, fatigue, cognitive dysfunction, depression, and anxiety^4–6^. Our recent findings suggest that autoimmunity may play a role in the symptoms of FM. Immunoglobulin G (IgG) antibodies from FM subjects, when transferred into mice, induced FM-like symptoms^7^ such as heightened sensitivity to painful stimuli and reduced intraepidermal nerve fiber density (IENFD), consistent with previous observations in FM patients^8^. Notably, the FM IgG was found to bind to both human and murine satellite glial cells (SGCs) in the dorsal root ganglia (DRGs) (anti-SGC IgG). Stressing the clinical relevance, the anti-SGC IgG levels from FM patients were associated with pain intensity and disease severity^9^.

Bile acids (BAs) are steroid acids essential for lipid absorption with inflammatory and immune functions^10,11^. They play significant roles in gastrointestinal disorders like bile acid diarrhea (BAD), often seen in subjects with diarrhea-predominant irritable bowel syndrome (IBS-D)^12,13^. Bile acids are cholesterol metabolites produced in the liver (primary BAs) that can be transported to the intestines and modified by the gut microbiota (secondary BAs). Minerbi et al. reported that the secondary BA α-muricholic acid (α-MCA) was depleted in overweight individuals with FM and was negatively associated with the widespread pain index and scores of the fibromyalgia impact questionnaire (FIQ)^14^. However, the levels of autoantibodies in FM or questionnaires used in clinical settings to assess depression or anxiety were not included in the previous study.

Short-chain fatty acids (SCFAs), another group of gut microbiota-derived metabolites, have been found altered in serum from FM subjects compared to HC^15^. Importantly, a recent study displayed that fecal transplantation from FM subjects into germ-free mice induces pain-like symptoms in the mice^16^. This was also seen to be associated with alterations in gut-derived metabolites^16^. However, to the best of our knowledge, associations between serum SCFA concentrations and FM symptoms have not been examined. Although SCFAs are implicated in neuropathic pain and autoimmune conditions, such as inflammatory bowel disease (IBD)^17,18^, their role in autoimmunity in FM remains unexplored.

This study investigated the associations between anti-SGC IgG levels and BAs and SCFAs concentrations in normal-weight FM subjects and healthy controls (HC). Furthermore, we examined how these associations relate to symptoms of FM, assessed with several established questionnaires to assess pain, depression, and other aspects of FM.

## Methods

### Study participants

The participants of this study were 35 FM subjects and 32 HC, non-overweight (body mass index (BMI) below 25) females aged 20–60 years from a larger cohort, which has been previously described^19–24^. The local ethical review committee has approved the study (no. 2014/1604-31/1), and the participants provided written informed consent. All methods are carried out in accordance with the Declaration of Helsinki.

Fibromyalgia subjects met both the 1990^25^ and 2011^26^ American College of Rheumatology (ACR) FM classification criteria, verified by a pain specialist. The participants were recruited between 2015 and 2017, prior to the publication of the revised 2016 ACR FM criteria^27^. Exclusion criteria were not speaking Swedish, hypertension (> 160/90 mmHg), pregnancy, autoimmune, pain, neurological, severe somatic or psychiatric disorders (other than FM), diabetes, smoking more than five cigarettes daily, substance abuse, and current use of antidepressant, anticonvulsant, or treatment for anxiety or depression. Participants also had to abstain from analgesics, nonsteroidal anti-inflammatory drugs (NSAIDs), or hypnotics for 48 h before participation. The HC did not fulfill the exclusion criteria above, were devoid of chronic pain, and did not take regular sleep medication, antidepressants, NSAIDs, analgesics, or anticonvulsants.

### Questionnaires

The number of months with pain (pain duration) and FM (FM duration), as well as total FIQ scores, were noted for the FM subjects. Pain intensity ratings using the visual analogue scale (VAS), Hospital Anxiety and Depression (HAD) Scale, and short form-36 health survey (SF-36) questionnaire were filled out by both FM subjects and HC. The FIQ assesses the disability of FM patients, where a higher score reflects a higher disability^28^. The pain intensity ratings were assessed with a VAS for weekly average pain (VASavr) and maximum pain during the last week (VASmax). The HAD scale assesses depression (HAD-D) and anxiety (HAD-A), where a higher score indicates a higher likelihood of the condition^29^. The subjects also filled in the short form-36 health survey (SF-36), where a low score reflects a lower health-related quality of life. Two summaries of this questionnaire were used for this study: the summary of the mental aspects of SF-36 (SF-36 MCS) and the physical aspects of SF-36 (SF-36 PCS). The SF-36 MCS summarizes the subcategories: emotional role, social functioning, mental health, and vitality. The SF-36 PCS summarizes general health, physical functioning, physical role, and bodily pain.

### Assessment of anti-SGC IgG levels

The anti-SGC IgG levels were estimated through immunocytochemistry. The protocol has been described previously^9^ and the anti-SGC IgG dataset has been published before^23^. All procedures were approved by Stockholm Norra Djurförsöksetiska nämnd (no. 4945 − 2018). All methods were carried out in accordance with relevant national and institutional guidelines and regulations for animal research. The study is reported in accordance with the ARRIVE guidelines. In brief, SGC-enriched cell cultures were obtained from DRGs harvested from adult female BALB/cAnNRj mice euthanized using 5% isoflurane followed by decapitation. Approximately 35–40 dorsal root ganglia were collected per preparation and dissociated enzymatically as previously described. The SGC cell cultures were incubated with either FM or HC serum IgG. Following fixation, cell cultures were first incubated with an SGC marker (glutamine synthesis rabbit IgG, Abcam ab73593) and then incubated with an IgG marker using anti-human IgG (AF594, Thermo Fisher A11014) and anti-rabbit IgG antibody (AF488, Thermo Fisher A11008). A customized machine-learning pipeline^30^ was then used to analyze the microscope images and determine the percentage of IgG binding to SGCs (IgG + SGC%), which assessed anti-SGC IgG levels for each sample. A more detailed summary of the method can be found in the Supplementary Material.

### Mass spectrometry

The concentrations of BAs and SCFAs were determined by liquid chromatography coupled with high-resolution mass spectrometry (LC-HRMS). Bile acids and SCFAs were analyzed separately using different experimental methods on the same analytical platform, Ultimate 3000 chromatography system interfaced with Q Exactive Orbitrap MS (Thermo Scientific, Waltham, MA, USA). All analyses were performed in negative ionization with a resolution of 70,000.

### Bile acids

Fifty µL methanol (MeOH) and 25 µL of a MeOH solution containing isotopically labeled internal standards (IS) were added to 25 µL serum from FM subjects and HC. The included BA IS are listed in the Supplementary Table S1. The samples were vortexed 15 s and left in a freezer at -20 °C for 60 min, followed by centrifugation at 21,100 RCF for 15 min at 4 °C. The supernatant was then transferred into HPLC vials and stored at -80 °C until further analysis.

The samples were injected (20 µL) on a reversed-phase HPLC column (Accucore C18 100 × 2.1 mm, 2.6 μm, Thermo Scientific). A 17.5 min long chromatographic program, including a gradient, was applied using the mobile phases H_2_O with 0.1% acetic acid and ten mM ammonium acetate and MeOH with 0.1% acetic acid and ten mM ammonium acetate. The flow rate was 0.6 mL/min, and the column temperature was 55 °C. A list of all authentic reference standards corresponding to endogenous BAs used to confirm BA identities are listed in Supplementary Table S1. Of note, the α-, β- and ω-isomers of MCA could not be chromatographically separated and are therefore reported as a sum. The HRMS analysis was performed in full scan mode, collecting data in the range of *m/z* 212.5–750 using ionization parameters previously described in detail^31^.

### Short-chain fatty acids

Serum samples (20 µL) were precipitated with 80 µL of cold methanol containing SCFA IS, listed in Supplementary Table S1. The samples were vortexed and centrifuged at 21,100 RCF for 15 min at 4°C. The supernatant was transferred to a new Eppendorf tube for derivatization with 3-nitrophenylhydrazine (25 mM), N-(3-Dimethylaminopropyl)-N’-ethylcarboimiide hydrochloride (EDC) (25 mM) and pridine (7%, w/w). The samples were shaken for 30 min at 40 °C. The derivatized samples were transferred to HPLC vials and diluted 5.8 times with 0.25% (v/v) formic acid in 1:1 MeOH: water. The samples were stored at -80 °C until further analysis.

Two µL of sample was injected on a reversed-phase HPLC column (Accucore C18 100 × 2.1 mm, 2.6 μm, Thermo Scientific). The two mobile phases contained 0.1% (v/v) formic acid in water and 1:9 isopropanol: MeOH, respectively. The HRMS analysis was performed in the full scan mode of *m/z* 100–1000 with two selected ion monitoring (SIM) windows: 3.9–4.9 min *m/z* 221–224 and 4.9–6.3 min *m/z* 235–238. A list of all authentic reference standards used to confirm SCFA identities is presented in Supplementary Table S1. The LC-HRMS method for SCFA analysis is described in detail in the Supplementary Material.

### Concentration calculations

Calibration curves were generated for SCFAs for which both the native compound and IS were available: acetic acid, propionic acid, 3-hydroxybutyric acid, butyric acid, isobutyric acid, 2-methylbutyric acid, isovaleric acid, valeric acid, and caproic acid. The concentrations of the remaining SCFAs and the BAs were determined through single-point calibration using the IS closest in retention time. The concentrations of acetic acid were adjusted for background levels in the calibrators.

### Statistical analysis

All statistical analyses were performed in R (version 4.3.3). Bile acid concentrations were summarized per class: nonconjugated primary, nonconjugated secondary, conjugated (glycine and taurine) primary, and conjugated (glycine and taurine) secondary. The total SCFA concentrations were also summarized. Linear regression models, adjusted for age and BMI, were used to compare groups with log_2_ transformed concentrations and summaries of BAs and SCFAs between FM subjects and HC and between FM subjects with high (≥ 50%) frequency of IgG binding to SGCs (IgG + SGC%) and low (< 50%) IgG + SGC%.

Hierarchical clustering was performed to assess correlations between the compounds, which revealed high dependencies between compounds. To account for multiple testing in the data analysis and the dependence of BA measurements within each of the BA classes and SCFAs, the significance threshold was adjusted to *P* ≤ 0.01.

The BA and SCFA concentrations of FM subjects were correlated with the demographic and clinical data collected. The correlation analysis for IgG + SGC% was performed with Pearson correlation on log_2_ transformed concentrations. The rest of the clinical data was correlated with Spearman rank correlation since they were not normally distributed. *P* ≤ 0.01 was considered statistically significant.

## Results

### Participant characteristics

**Fig. 1:**
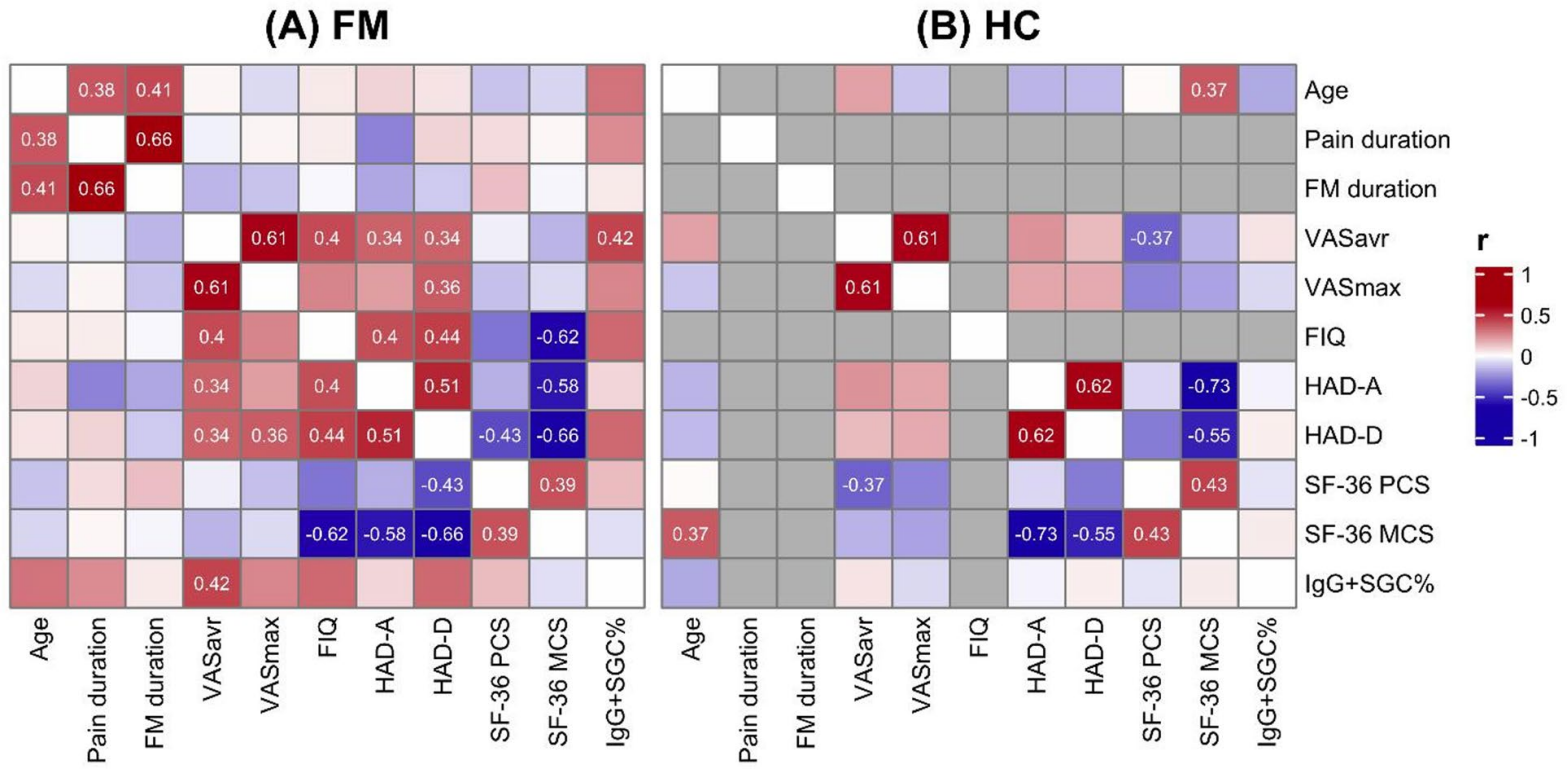
Pairwise correlations among clinical and demographic measures in fibromyalgia (FM) (**A**) and healthy controls (HC) (**B**). Each cell represents a Spearman correlation coefficient (r), with red indicating positive correlations and blue indicating negative correlations. Only correlations with *P* < 0.05 are shown. VASavr: average weekly pain intensity on a Visual Analogue Scale, VASmax: maximum pain intensity during the past week on a Visual Analogue Scale, FIQ: Fibromyalgia Impact Questionnaire, HAD-A: anxiety scores from the Hospital Anxiety and Depression Scale, HAD-D: depression scores from the Hospital Anxiety and Depression Scale, SF-36 MCS: mental component of the Short-Form-36 Healthy Survey, SF-36 PCS: physical component of the Short-Form-36 Healthy Survey.

Subjects with FM exhibited significantly higher pain ratings on a visual analogue scale (VAS), higher depression and anxiety scores on a Hospital Anxiety and Depression (HAD) Scale, and lower scores of the physical (SF-36 PCS) and mental (SF-36 MCS) components of the short form-36 health survey compared to HC (Table 1). Notably, FM subjects also had elevated levels of anti-SGC IgG (IgG + SGC%). There were no differences in age or BMI between any groups. Pairwise correlation analyses were conducted for parameters in both FM subjects and HC to explore relationships between demographic and clinical data (Fig. 1). Anti-SGC IgG levels positively correlated with higher pain intensities in FM subjects. Overall, these findings indicate a poorer quality of life and increased anti-SGC IgG levels in FM subjects compared to HC.

**Table 1:** The descriptive statistics of the fibromyalgia (FM) subjects and healthy controls (HC) are presented as means ± standard deviation. The individuals were divided into HC or FM subjects. The FM subjects were then further divided into low (< 50%) or high (≥ 50%) frequency of immunoglobulin G binding to satellite glial cells (IgG + SGC%). The pain and FM duration (months) and fibromyalgia impact questionnaire (FIQ) scores were only recorded for FM subjects.

	HC	FM	FM low IgG + SGC%	FM high IgG + SGC%	FM vs. HC *P*	High vs. low IgG + SGC% *P*
Samples (*n*)	32	35	19	16	NA	NA
Age (year)	47.8 ± 7.7	47.1 ± 8.4	45.2 ± 8.5	49.4 ± 7.8	0.846	0.149
BMI (kg/m^2^)	22.1 ± 1.5	22.5 ± 1.5	22.5 ± 1.4	22.5 ± 1.7	0.33	0.895
Pain duration (months)	NA	166.8 ± 89.1	138 ± 77.5	201 ± 92.1	NA	0.038*
FM duration (months)	NA	117.2 ± 78.5	104.7 ± 73.6	131.9 ± 83.9	NA	0.678
VASavr (mm)	4.9 ± 6.4	57.1 ± 18.2	50.6 ± 16.9	64.9 ± 17	< 0.001*	0.033*
VASmax (mm)	14.3 ± 21.1	75.7 ± 14.2	72.2 ± 16	80 ± 10.7	< 0.001*	0.154
FIQ	NA	61.4 ± 14	57.8 ± 12.2	65.8 ± 15.2	NA	0.154
HAD-A	2.9 ± 3	7.7 ± 4.7	7.6 ± 4.1	7.7 ± 5.4	< 0.001*	0.702
HAD-D	1.3 ± 1.6	6.6 ± 4.2	5.5 ± 3.9	7.9 ± 4.3	< 0.001*	0.07
SF-36 PCS	93.3 ± 6.5	39.6 ± 14.4	38.7 ± 9.6	40.6 ± 18.9	< 0.001*	0.974
SF-36 MCS	86.8 ± 12.1	52.3 ± 19.9	53.9 ± 14.8	50.5 ± 25.1	< 0.001*	0.96
IgG + SGC% (%)	31 ± 18.8	48.5 ± 25.6	28.3 ± 13.5	72.4 ± 11.9	0.004*	< 0.001*

*: *P* < 0.05, VAS: pain intensity ratings in a Visual Analogue Scale, VASavr: average weekly pain intensity, VASmax: maximum pain intensity during the past week, HAD: Hospital anxiety (HAD-A) and depression (HAD-D) scale, SF-36 MCS: mental component of short form-36 health survey, SF-36 PCS: physical component of short form-36 health survey.

### Secondary bile acids are elevated in FM subjects compared to HC

**Fig. 2:**
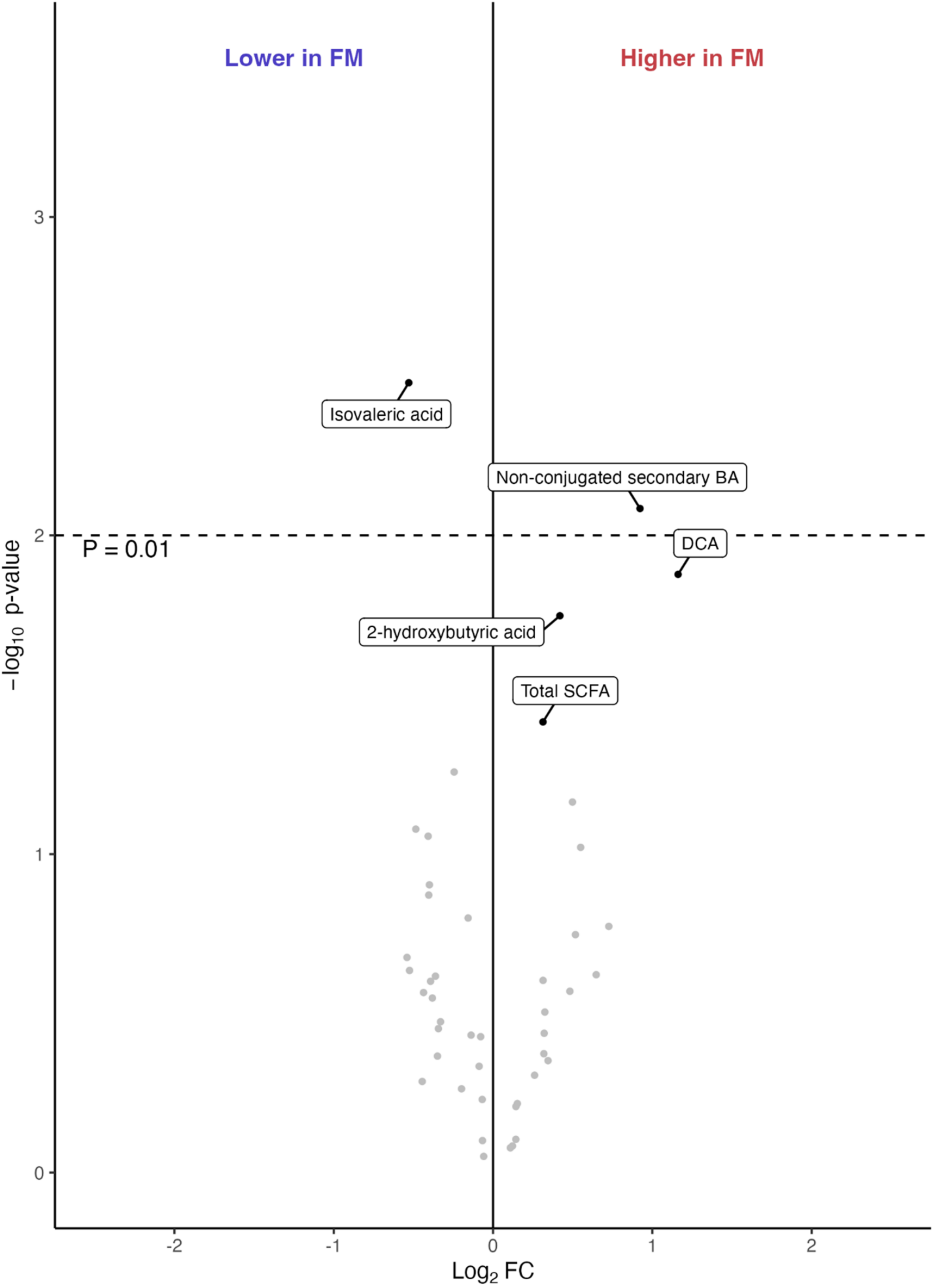
Volcano plot of bile acids (BAs), short-chain fatty acids (SCFAs), and the class summaries comparing fibromyalgia (FM) subjects and healthy controls (HC). The x-axis shows log_2_ fold change (FC) and the y-axis shows -log_10_ p-values from linear regression adjusted for age and body mass index. Annotated compounds have *P* < 0.05. The dashed line indicates the threshold for significance after adjustment for multiple testing (*P* ≤ 0.01). Non-conjugated secondary BAs were significantly increased in FM subjects compared to HC, while deoxycholic acid (DCA), the most common secondary BA, did not reach significance. Isovaleric acid was instead significantly decreased in FM subjects.

Hierarchical clustering and pairwise correlations showed strong within-class dependencies among BAs and SCFAs (i.e., metabolites clustered by biochemical subclass), in both FM and HC (Supplementary Fig. S1). When comparing BA concentrations between FM subjects and HC, deoxycholic acid (DCA) showed a nominal group difference, but this did not remain significant after correction for multiple testing (*P* = 0.013) (Table 2; Fig. 2). In line with this trend, the total concentration of non-conjugated secondary BAs was significantly (*P* = 0.008) higher in FM subjects than in HC (Table 3; Fig. 2). This indicates an altered BA profile in FM subjects, characterized by increased microbially produced secondary BAs.

**Table 2:** Certain glycine (Gly)-conjugated bile acids (BAs) are elevated in fibromyalgia subjects with a high frequency of immunoglobulin G binding to satellite glial cells (IgG + SGC%). Short-chain fatty acid (SCFA) isovaleric acid was significantly elevated in FM subjects compared to healthy controls (HC). The concentrations are presented as mean (min - max) in micromolar. The log_2_ transformed fold change (FC) was extracted from the linear regression models results, adjusted for age and body mass index.

Compound	Shortened name	Class	Concentration (µM)		Linear regression			
			FM	HC	FM - HC		FM high - low IgG + SGC%	
					Log_2_ FC	P	Log_2_ FC	P
Cholic acid	CA	Non-conjugated primary BA	0.0862 (0.00118–1.21)	0.0356 (0.00167–0.533)	0.12	0.825	1.94	0.025
Chenodeoxycholic acid	CDCA	Non-conjugated primary BA	0.0931 (0.00112–1.71)	0.0309 (0.00191–0.359)	0.11	0.836	2.09	0.015
Deoxycholic acid	DCA	Non-conjugated secondary BA	0.174 (0.00217–1.19)	0.0753 (0.00286–0.362)	1.16	0.013	1.46	0.017
Hyocholic acid	HCA	Non-conjugated secondary BA	0.00135 (3.37e-06–0.0127)	0.000823 (7.14e-05–0.00508)	0.35	0.445	1.55	0.036
Hyodeoxycholic acid	HDCA	Non-conjugated secondary BA	0.0242 (0.00248–0.0906)	0.0196 (0.00141–0.0853)	0.55	0.095	0.65	0.107
Lithocholic acid	LCA	Non-conjugated secondary BA	0.00532 (5.36e-05–0.0349)	0.00501 (0.000215–0.0363)	0.26	0.495	0.86	0.077
Muricholic acid (nonspecific)	MCA	Non-conjugated secondary BA	0.00138 (5.64e-06–0.00998)	0.000583 (1.57e-05–0.00403)	0.73	0.169	1.8	0.025
Murideoxycholic acid	MDCA	Non-conjugated secondary BA	0.0144 (0.00112–0.045)	0.0121 (0.00136–0.102)	0.33	0.313	0.21	0.668
Ursodeoxycholic acid	UDCA	Non-conjugated secondary BA	0.00891 (0.000591–0.07)	0.00532 (0.000829–0.0396)	0.32	0.365	1.1	0.049
Glycocholic acid	Gly-CA	Gly-conjugated primary BA	0.297 (0.0133–1.5)	0.386 (0.0412–1.76)	-0.39	0.251	1.07	0.034
Glycochenodeoxycholic acid	Gly-CDCA	Gly-conjugated primary BA	0.453 (0.0829–1.17)	0.579 (0.168–2.09)	-0.41	0.088	0.79	0.035
Taurocholic acid	Tau-CA	Tau-conjugated primary BA	0.0594 (0.00285–0.733)	0.0816 (0.00401–0.548)	-0.54	0.211	0.78	0.189
Taurochenodeoxycholic acid	Tau-CDCA	Tau-conjugated primary BA	0.182 (0.0204–1.37)	0.219 (0.0283–0.923)	-0.33	0.336	0.48	0.336
Glycodeoxycholic acid	Gly-DCA	Gly-conjugated secondary BA	0.286 (0.011–1.24)	0.23 (0.00769–1.5)	0.52	0.179	1.18	0.02
Glycohyocholic acid	Gly-HCA	Gly-conjugated secondary BA	0.00916 (0.00103–0.0275)	0.0127 (0.00197–0.0503)	-0.48	0.083	0.87	0.051
Glycohyodeoxycholic acid	Gly-HDCA	Gly-conjugated secondary BA	0.0103 (2.54e-05–0.0297)	0.00691 (5.11e-05–0.021)	0.65	0.239	2.09	0.006*
Glycolithocholic acid	Gly-LCA	Gly-conjugated secondary BA	0.0155 (7.29e-05–0.0751)	0.0156 (0.000519–0.0597)	-0.35	0.431	1.17	0.094
Glycolithocholic acid 3-sulfate	Gly-LCA 3-S	Gly-conjugated secondary BA	0.0503 (0.00354–0.169)	0.0666 (0.00195–0.402)	-0.36	0.242	1.24	0.001*
Glycoursodeoxycholic acid	Gly-UDCA	Gly-conjugated secondary BA	0.0696 (0.00513–0.289)	0.0783 (0.0178–0.493)	-0.2	0.546	0.57	0.288
Taurodeoxycholic acid	Tau-DCA	Tau-conjugated secondary BA	0.138 (0.00805–0.751)	0.119 (0.00213–0.677)	0.48	0.27	0.53	0.323
Taurohyocholic acid	Tau-HCA	Tau-conjugated secondary BA	0.00284 (0.000307–0.0181)	0.00519 (2.12e-05–0.0282)	-0.52	0.232	0.43	0.436
Taurohyodeoxycholic acid	Tau-HDCA	Tau-conjugated secondary BA	0.00343 (2.48e-05–0.0206)	0.00379 (4.26e-05–0.0144)	-0.44	0.518	1.17	0.226
Taurolithocholic acid	Tau-LCA	Tau-conjugated secondary BA	0.00653 (0.000141–0.043)	0.0077 (0.000182–0.0319)	-0.44	0.272	0.47	0.393
Tauroursodeoxycholic acid	Tau-UDCA	Tau-conjugated secondary BA	0.00397 (6.3e-05–0.0208)	0.00502 (0.000151–0.044)	-0.06	0.89	0.02	0.972
	Acetic acid	C2 SCFA	94 (58.9–127)	99.1 (57.6–157)	-0.08	0.374	-0.13	0.278
	Propionic acid	C3 SCFA	2.62 (1.46–4.69)	2.77 (1.12–5.99)	-0.07	0.59	-0.05	0.775
	2-Hydroxybutyric acid	C4 SCFA	116 (32.6–283)	82.7 (28.8–176)	0.42	0.018	-0.28	0.306
	3-Hydroxybutyric acid	C4 SCFA	153 (20.9–670)	81.8 (24.9–205)	0.5	0.069	-0.83	0.079
	Acetoacetic acid	C4 SCFA	12 (0.861–52.9)	7.27 (2.72–22.8)	0.31	0.249	-0.68	0.16
	Butyric acid	C4 SCFA	6.79 (3.57–11.7)	7.33 (3.91–16.4)	-0.09	0.464	-0.1	0.545
	Isobutyric acid	C4 SCFA	0.993 (0.585–1.75)	1.11 (0.563–2.04)	-0.16	0.159	-0.26	0.106
	2-Methylbutyric acid	C5 SCFA	0.254 (0.062–0.485)	0.304 (0.11–0.524)	-0.24	0.055	0.12	0.496
	Isovaleric acid	C5 SCFA	0.537 (0.191–1.31)	0.766 (0.265–1.49)	-0.53	0.003*	-0.05	0.848
	Valeric acid	C5 SCFA	0.218 (0.092–0.403)	0.245 (0.064–0.49)	-0.14	0.37	-0.1	0.617
	Caproic acid	C6 SCFA	0.389 (0.0055–0.823)	0.486 (0.0055–1.09)	-0.34	0.353	-0.25	0.652

*: Significant after adjustment (*P* ≤ 0.01), Tau: taurine, Gly: glycine.

**Table 3:** Fibromyalgia (FM) subjects had increased concentrations of non-conjugated secondary bile acids (BAs) compared to healthy controls (HC). Interestingly, FM subjects with a high (≥ 50%) frequency of immunoglobulin G binding to satellite glial cells (IgG + SGC%) had elevated total BA levels, compared to FM subjects with low IgG + SGC%. The concentrations are presented as mean (min - max) in micromolar. The log_2_ transformed fold change (FC) is extracted from the linear regression model results for the summarized groups for short-chain fatty acids (SCFAs) and BAs, adjusted for age and BMI.

Class	Concentration (µM)		Linear regression			
	FM	HC	FM - HC		FM High - Low IgG + SGC%	
			Log_2_ FC	*P*	Log_2_ FC	*P*
Total SCFA	386.594 (144.82–1028.08)	283.886 (166.499–544.592)	0.31	0.038	-0.42	0.11
Total BA	1.996 (0.352–6.288)	2 (0.36–7.44)	-0.07	0.794	1.13	0.003*
Non-conjugated primary BA	0.179 (0.002–2.927)	0.067 (0.005–0.893)	0.14	0.787	2.03	0.017
Non-conjugated secondary BA	0.236 (0.028–1.312)	0.122 (0.012–0.473)	0.92	0.008*	1.06	0.018
Conjugated primary BA	0.991 (0.137–4.693)	1.265 (0.261–4.469)	-0.4	0.134	0.87	0.034
Gly-conjugated primary BA	0.75 (0.096–2.593)	0.965 (0.229–3.851)	-0.4	0.125	0.9	0.025
Tau-conjugated primary BA	0.241 (0.023–2.101)	0.301 (0.032–1.3)	-0.38	0.283	0.56	0.266
Conjugated secondary BA	0.595 (0.063–2.364)	0.549 (0.074–2.557)	0.15	0.607	0.96	0.022
Gly-conjugated secondary BA	0.441 (0.038–1.545)	0.409 (0.067–2.183)	0.14	0.62	1.04	0.012
Tau-conjugated secondary BA	0.155 (0.013–0.818)	0.14 (0.006–0.73)	0.32	0.423	0.5	0.335

*: Significant after adjustment (*P* ≤ 0.01), Tau: taurine, Gly: glycine.

### Fibromyalgia subjects with high anti-SGC IgG levels have increased bile acid concentrations

**Fig. 3:**
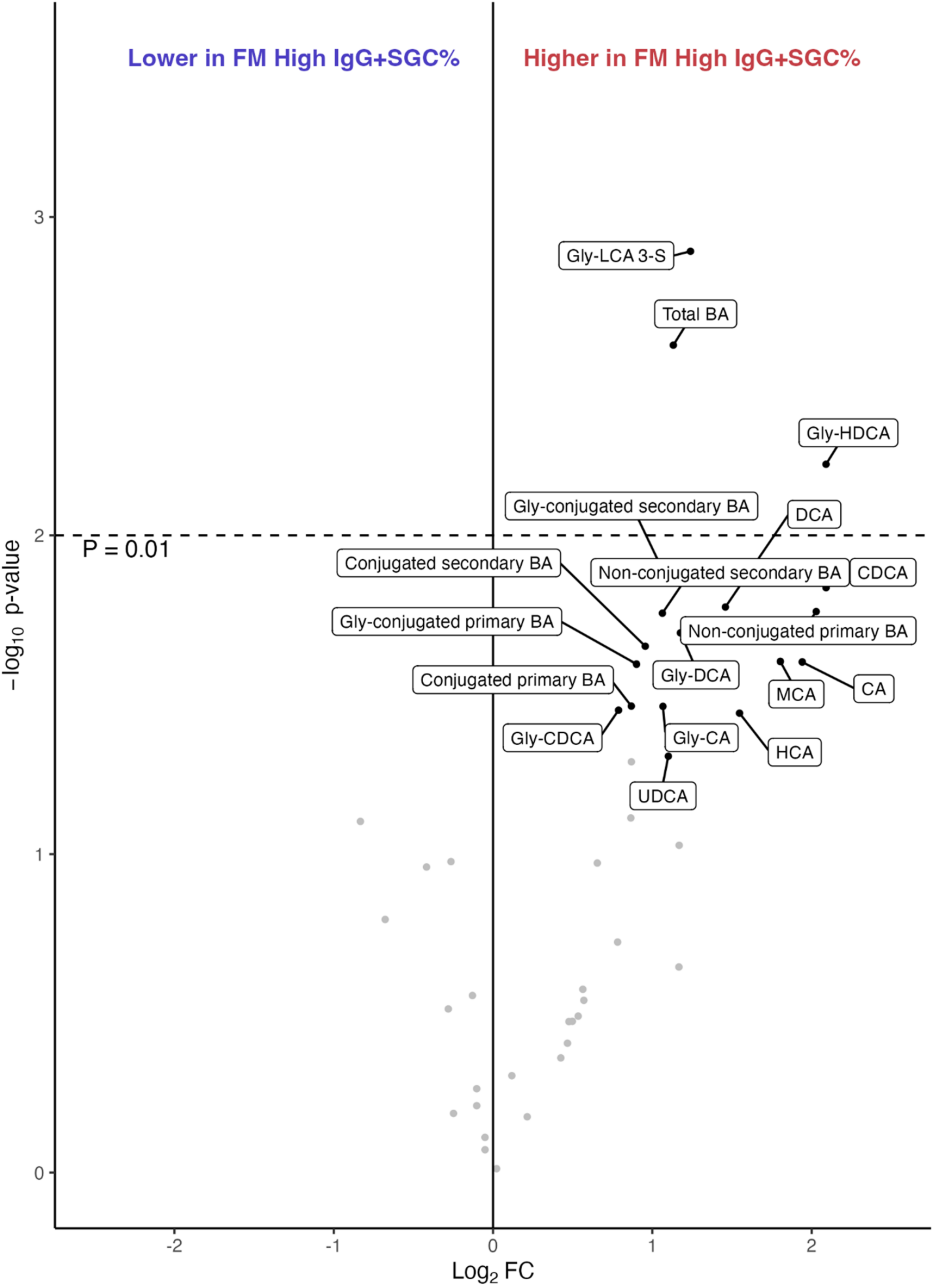
Volcano plot of bile acids (BAs), short-chain fatty acids (SCFAs), and the class summaries comparing fibromyalgia (FM) patients with high and low frequency of immunoglobulin G binding to satellite glial cells (IgG + SGC%). The x-axis shows log_2_ fold change (FC) and the y-axis shows -log_10_ p-values from linear regression adjusted for age and BMI. Annotated compounds have *P* < 0.05. The dashed line indicates the threshold for significance after adjustment for multiple testing (*P* ≤ 0.01). Total BAs were found significantly increased in FM subjects with high IgG + SGC%, with specific BAs showing the same trend. Abbreviations – CA: cholic acid, CDCA: chenodeoxycholic acid, DCA: deoxycholic acid, Gly: glycine, HCA: hyodeoxycholic acid, HDCA: hyodeoxycholic acid, MCA: muricholic acid, LCA: lithocholic acid, UDCA: ursodeoxycholic acid.

The most pronounced differences in BA concentrations were observed between FM subjects with high *versus* low IgG + SGC%. Overall, FM subjects with high IgG + SGC% had significantly higher levels of BAs compared to those with low IgG + SGC% (Table 2; Fig. 3). Several individual BAs were nominally elevated in the FM subjects with high IgG + SGC% (*P* < 0.05), including chenodeoxycholic acid (CDCA), cholic acid (CA), deoxycholic acid (DCA), hyocholic acid (HCA), non-specific muricholic acid (MCA), ursodeoxycholic acid (UDCA), glycine conjugated CDCA (Gly-CDCA), Gly-CA, Gly-DCA, Gly-hyodeoxycholic acid (Gly-HDCA), and Gly-lithocholic acid 3-sulfate (Gly-LCA 3-S). Of note, none of the taurine (Tau)-conjugated BAs showed differences in FM with high IgG + SGC% compared to low. After adjustment for multiple testing, Gly-LCA-3-S (*P* = 0.001) and Gly-HDCA (*P* = 0.006) remained significantly elevated.

Summarized by class, FM subjects with high IgG + SGC% had elevated levels of higher total, non-conjugated, and Gly-conjugated BAs, but no difference was seen for Tau-conjugated BAs (Table 3; Fig. 3). The total BA concentrations remain statistically significant after adjustment (*P* = 0.003). These findings suggest that FM subjects with high anti-SGC IgG levels have elevated concentrations of BAs compared to those with low anti-SGC IgG levels.

### Conjugated bile acids are associated with poorer mental well-being and increased disease severity in fibromyalgia subjects

**Fig. 4:**
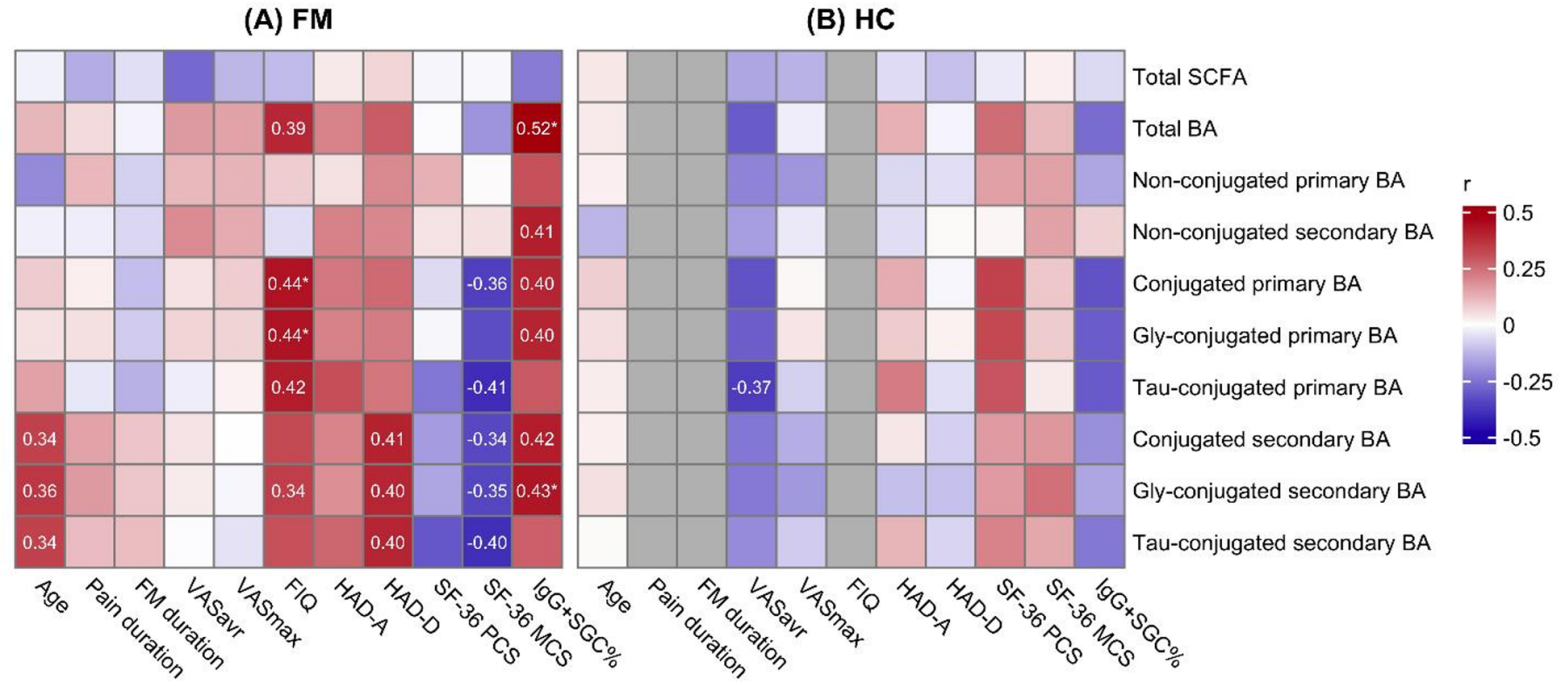
Correlations between summarized bile acid (BA) classes and short-chain fatty acids (SCFAs) and clinical measures in fibromyalgia (FM) subjects (**A**) and healthy controls (HC; **B**). The clinical measures correlated were: pain ratings on a Visual Analogue Scale (VAS), the impact of FM assessed with total scores of the Fibromyalgia Impact Questionnaire (FIQ), anxiety and depression scores from the Hospital Anxiety and Depression Scale (HAD-A and HAD-D), physical and mental summaries of the short-form-36 health survey (SF-36 PCS and MCS) assessing health-related quality of life. Additionally, the frequency of IgG binding to satellite glial cells (IgG + SGC%) was correlated. A red color indicates a positive correlation, a blue color indicates a negative correlation, and a grey color indicates not applicable. The numbers shown are the correlation coefficients for correlation with *P* < 0.05, and statistical significance after adjustment (*P* ≤ 0.01) is indicated with *. A positive trend was seen between BA classes and IgG + SGC%, with total BA and glycine (Gly)-conjugated secondary BA levels being significantly associated (*P* < 0.01). Additionally, conjugated and Gly-conjugated primary BAs were associated with the impact of FM (higher FIQ scores). Abbreviations - Tau: taurine, VASavr: average weekly pain intensity, VASmax: maximum pain intensity during the past week.

A pairwise correlation analysis was conducted between each clinical parameter and the compound concentrations in FM subjects to investigate potential connections between altered concentrations and FM symptoms. Conjugated BAs were found to correlate with poorer mental well-being. After adjustment for multiple testing, Gly-LCA showed a positive association with anxiety scores (HAD-A) and a negative association with mental well-being (SF-36 MCS), and Gly-LCA-3-S was positively associated with depression scores (HAD-D) (Supplementary Fig. S2). At the class level, nominal associations were found for conjugated primary and secondary BAs with SF-36 MCS and FIQ (Fig. 4). However, only the association between FIQ and Gly-conjugated primary BAs remained significant following correction.

Secondary Gly-conjugated BAs also demonstrated significant associations with autoantibodies. Specifically, Gly-LCA and Gly-LCA-3-S were positively associated with IgG + SGC%, and these associations remained significant after adjustment (Supplementary Fig. S2, Supplementary Table S2). Tau-conjugated BAs did not show corresponding associations (Fig. 4, Supplementary Table S3). Together, these findings indicate that Gly-conjugated secondary BAs are associated with both psychological symptom severity and anti-SGC IgG.

### Short-chain fatty acid concentrations are altered in FM subjects but not associated with symptom severity

Two SCFAs, isovaleric acid and 2-hydroxybutyric acid, exhibited nominal differences between individuals with FM and HC. After adjustment, only the decrease in isovaleric acid remained significant (*P* = 0.003) (Table 2; Fig. 2). However, when summarized, no significant differences were observed (Table 3). In FM subjects, trends were observed between isobutyric acid and VASmax, as well as 2-methylbutyric acid and HAD-A, but were not significant after adjustment. No significant correlations between isovaleric acid, 2-hydroxybutyric acid, or total SCFA concentrations were observed with clinical data or FM symptoms (Fig. 4, Supplementary Fig. S2, Supplementary Tables S2 and S3). Taken together, FM subjects have decreased isovaleric acid concentrations compared to HC, but these changes did not correlate with FM symptoms.

## Discussion

This study explored the potential connection between autoimmune mechanisms in FM, changes in BAs and SCFAs, and their associations with FM symptoms. Our findings show elevated levels of secondary BAs in FM subjects compared to HC. Notably, FM subjects with higher anti-SGC IgG levels exhibited elevated total BA levels compared to those with lower anti-SGC IgG levels. Individual BA concentrations, including Gly-CA, Gly-HDCA, and Gly-LCA, were associated with increased disease severity, poor mental well-being, and increased depressive symptoms. These findings suggest that BAs may contribute to both the psychological symptoms and autoimmune aspects of FM.

### Secondary bile acids in fibromyalgia

Bile acids are humans’ primary product of cholesterol catabolism^32^. There are conflicting reports on total cholesterol levels in FM subjects. Some studies have reported higher total cholesterol levels^33–35^, while other studies have found no significant difference between FM subjects and HC^36,37^. Our analysis shows a notable increase in secondary BAs in FM subjects, while the total BA concentrations are unchanged compared to HC. Rather than an increased cholesterol breakdown, we therefore hypothesize that there may be a shift towards secondary BA synthesis. This shift may be attributed to the previously documented gut microbiome alteration in FM subjects^15,38–40^ or due to an increased intestinal permeability observed in FM subjects^41,42^.

Previously, Minerbi et al. reported decreased α-MCA in FM subjects compared to HC^14^. In our analysis, we did not observe a significant difference in MCA concentrations. However, our MCA measurement represents the combined signal of the α-, β- and ω-isomers, which could not be chromatographically resolved, and therefore direct comparisons with α-MCA specifically cannot be made. In addition, the cohorts differed in BMI distribution (mean BMI 22.5 in our study vs. 29.4 in Minerbi et al.)^14^, which is relevant because BMI can influence BA synthesis and composition.

Our results showed an increase in DCA concentrations in FM subjects compared to HC, which can potentially contribute to inflammation. Low-grade inflammation has been implicated in FM^43^ and IBS^44^. Repetitive instillation of DCA in colon induces low-grade colonic inflammation, leading to persistent visceral hypersensitivity and referred pain in rats^45^. Thus, there may be a connection between BAs and low-grade inflammation in FM. The link to IBS is particularly interesting, as both FM and IBS are comorbid nociplastic pain conditions^6^ that may share underlying alterations in gut-brain axis function and BA metabolism. However, Minerbi et al. previously reported that BA differences in FM were largely independent of IBS status^14^. Because IBS was not assessed in the present study, we cannot exclude the possibility that part of the observed BA differences reflects undetected IBS comorbidity. Future studies, including a systematic assessment of gastrointestinal symptoms, will be important to clarify these relationships.

### Conjugated bile acids and depression

Fibromyalgia subjects are known to have an increased risk of mood disorders, such as depression^46^. Our results show a clear association between BAs and psychological symptoms, indicating a connection between BA metabolism and poor mental health in FM subjects. Dysregulation of BAs has been shown in individuals with major depressive disorder (MDD), where increased levels of 23-nordeoxycholic acid, a DCA derivative, were positively correlated with depression scores^47^. Interestingly, BA precursors (cholesterol derivatives) have also been found to correlate with depression scores, but not other symptoms, in Parkinsonism^48^.

In both IBD and MDD, alteration of gut microbiome composition and altered secondary BA metabolism have been associated with anxiety and depression scores^49,50^. Additionally, a recent study by Nhu and colleagues further highlighted the role of gut-brain axis disruption, possibly tied to BA metabolism, in psychological symptoms associated with FM, rather than pain^40^. Our results extend this understanding by showing that conjugated primary and secondary BAs are associated with mental health outcomes. However, since hepatic primary BAs show the same associations with mental health outcomes as the microbially produced secondary BAs, changes in the gut microbiome alone may not fully explain the connection. It is therefore plausible that the alterations in BA metabolism may contribute directly or indirectly to depressive symptoms in FM, as proposed in other diseases like MDD and IBD.

One potential mechanistic explanation for the connection to poor mental well-being involves signaling through the farnesoid X receptor (FXR), essential in BA homeostasis. This receptor has a known affinity for both primary and secondary BAs, including conjugated BAs, which may influence various physiological pathways linked to mental health^10,51^. A study in rats showed that stress increased FXR expression in the brain, leading to depressive-like symptoms^52^. Moreover, FXR knockout mice exhibited increased levels of BAs in serum and brain tissue, along with disruption in neurotransmitter pathways in different brain regions^53^. In FM patients, increased expression of FXR pathways has been reported^54^, with the authors suggesting that this increased activation of FXR pathways may be a response to chronic inflammation. However, as noted by Chen and colleagues^52^, this increase in FXR signaling could also be connected to depressive symptoms in FM. Further research into the relationship between FXR and BA signaling could shed light on the mechanisms underlying depressive symptoms in FM.

Another BA receptor, Takeda G protein-coupled receptor 5 (TGR5), has also been associated with anti-depressive symptoms, as TGR5 knockout mice exhibit anxiety and depression-like behaviors^55^. These behaviors were further linked to the gut microbiota, as fecal transplants from TGR5 knockout mice into healthy mice induced similar anxiety- and depression-like symptoms^55^. Taken together, these findings suggest that BAs and their receptors play a significant role in mental health, with potential implications for both MDD and FM. Therefore, further studies should clarify the functional involvement of FXR and TGR5 in order to expand on this hypothesis.

### Autoimmunity and bile acids

Previous studies have shown alterations in BA concentrations in autoimmune diseases, such as type 1 diabetes, multiple sclerosis, IBD, and autoimmune hepatitis^49,56–59^. This is particularly interesting, as our findings reveal a strong association between BA levels and the presence of autoantibodies binding to DRG SGCs in FM.

Bile acids can induce visceral hypersensitivity through FXR activation, increasing the expression of transient receptor potential vanilloid 1 (TRPV1) in DRGs^60^. Deoxycholic acid can directly and indirectly stimulate neurons in the colon and DRGs^61^. In a mouse model of peripheral neuropathic pain, DCA increased neuronal hyperexcitability dependent on TGR5 in DRGs^62^. However, BAs can also induce analgesic effects by binding to TGR5 on DRG neurons^63^. These findings highlight the dual anti- and pro-nociceptive roles of BAs, likely regulated via their receptor interactions. Our results show that DCA and ten other BAs are present at higher concentrations in FM subjects with high anti-SGC IgG levels than those with lower levels. These striking findings, along with the close proximity of autoantibody binding and BA activity, suggest a potential overlap or direct interactions between the two. The strong association between anti-SGC IgG and BAs in our study suggests that BAs may contribute to autoimmune mechanisms in FM.

When we further investigated conjugated BAs and their associations with anti-SGC IgG levels, we found that the association depended on the specific amino acid used for conjugation. Glycine-conjugated primary and secondary BAs significantly correlated with anti-SGC IgG levels, whereas the Tau-conjugated BAs showed no such correlations. In humans, Gly-conjugation is the most common BA conjugation, occurring at a 3:1 ratio compared to Tau-conjugation^64,65^. Animal studies have shown differences in affinity to TGR5 based on whether BAs are conjugated with glycine, taurine or are non-conjugated, with Tau-conjugated BAs acting as the most potent agonist^66^. Although the role of BA conjugation in chronic pain is currently unknown, our results suggest it may be influential on mental well-being.

### Short-chain fatty acid metabolism in fibromyalgia

Isovaleric acid is produced from leucine through microbial actions^67^. The SCFA-synthesizing bacterial species *Bifidobacterium* has been reported to correlate with isovaleric acid levels in fecal samples of young children^68^, and its abundance is reduced in FM subjects compared to controls^38^. Interestingly, FM subjects also have increased serum concentrations of leucine compared to HC^69^, which contrasts with the lower concentrations of isovaleric acid we see in FM subjects. This discrepancy suggests that the reduced levels of isovaleric acid in FM may be driven by changes in the gut microbiota rather than a decreased availability of leucine.

During oxidative stress, the glutathione synthesis rate increases, which produces 2-hydroxybutyric acid as a byproduct^71^. The elevated levels of 2-hydroxybutyric acid observed in FM subjects compared to HC may reflect the previously documented heightened oxidative stress in FM^72^. Additional research into SCFA disease-related mechanisms in FM is warranted, particularly since no significant associations with FM symptoms were identified.

### Limitations

The presented findings indicate only associations and do not establish causal relationships. Additionally, the study participants were not fasting prior to serum collection, and various lifestyle factors were not recorded, which could potentially influence the serum concentrations of SCFA and BA. Bile acid concentrations can be influenced by diurnal variation and fasting status^76,77^, which increases interindividual variability and may have obscured some differences. The modest sample size increases the risk of type I/II errors, and the restriction to non-overweight participants may also limit the generalizability of the findings. This study also only included females, and further studies are required to investigate if these results are similar in males. These factors highlight the importance of replication in larger, independent cohorts that include more diverse populations. Lastly, the study did not assess comorbid IBS, which may also impact the results.

## Conclusion

Our results reveal a strong correlation between BAs and anti-SGC IgG levels in individuals with FM. Notably, conjugated BAs are also associated with poorer mental well-being. This suggests that a disturbed BA circulation in FM may negatively affect mental health and contribute to depressive symptoms, highlighting an essential aspect of the condition that may be linked to the gut-brain axis and may offer pathways toward personalized treatment approaches tailored to specific subgroups of FM patients.

## Supplementary Information

Below is the link to the electronic supplementary material.

Supplementary Material 1

## Data Availability

Due to the Swedish Patient Data law and the conditions of approval from the Swedish Ethical Review Authority, individual-level data cannot be openly shared. Mass spectrometry data are available upon reasonable request to the corresponding author, Kim Kultima.
