# Supplementary material for: Microbially produced bile acids are associated with increased IgG autoantibodies and poorer mental wellbeing in fibromyalgia
Source: Sci Rep. 2026 Feb 24;16:7735. doi: 10.1038/s41598-026-40781-3 (PMC12949243; doi:10.1038/s41598-026-40781-3)
Supplement: Supplementary file 1 — Supplementary Material 1 [file 41598_2026_40781_MOESM1_ESM.docx]

Microbially produced bile acids are associated with increased IgG autoantibodies and poorer mental wellbeing in fibromyalgia

Jenny E. Jakobsson^a,b^, Henrik Carlsson^a^, Ida Erngren^a^, Joana Menezes^c^, Emerson Krock^c,1^, Matthew A. Hunt^c^, Jeanette Tour Sohlin^d^, Asma Al-Grety^a^, Katalin Sandor^c^, Eva Kosek^b,d^, Camilla I. Svensson^c^, Kim Kultima^a,c*^

^a^ Department of Medical Sciences, Uppsala University, Uppsala, Sweden.

^b^ Department of Surgical Sciences, Uppsala University, Uppsala, Sweden.

^c^ Department of Physiology and Pharmacology, Center for Molecular Medicine, Karolinska Institutet, Stockholm, Sweden.

^d^ Department of Clinical Neuroscience, Karolinska Institutet, Stockholm, Sweden.

^1^ Present affiliation: Faculty of Dental Medicine and Oral Health Sciences, Alan Edwards Centre for Research on Pain, McGill University, Montreal, Canada.

^*^ Corresponding author. Address: Department of Medical Sciences, Clinical Chemistry, Uppsala University, Akademiska sjukhuset entrance 61 3rd floor, 751 85 Uppsala, Sweden. Telephone number: +46 186114248. Institutional URL: [https://www.medsci.uu.se/](https://www.medsci.uu.se/?languageId=3). Email: kim.kultima@medsci.uu.se.

# Supplementary materials

# Supplementary methods

## Assessment of anti-SGC IgG levels

Blood samples from FM subjects and HC were collected from the median antecubital vein to a BD vacutainer® STT II tube. The serum was collected by aliquoting the supernatant after blood centrifugation at 2500 rpm for 10 minutes after being kept at room temperature for 40 minutes and then stored at –80°C. The experimenters were blinded to sample belonging during preparation and imaging. A primary cell culture of SGCs was prepared from DRGs harvested from adult female BALB/cAnNRj mice (approved by Stockholm Norra Djurförsöksetiska nämnd). The DRGs were separated by gentle shaking at 37℃ with a papain solution for 30 min, followed by collagenase/dispase solution for an additional 30 min. The cells were resuspended in F12 media with 10% calf serum and 1x penicillin-streptomycin. The cell culture was then triturated, filtered through a 100 μm cell strainer, and supplied to a Nunc™ Lab-Tek™ chamber slides. After 1.5 h, the supernatant was removed to discard non-SGCs. The SGCs were allowed a recovery period overnight in a 5% CO2 incubator at 37℃.

The following day, the frequency of IgG binding to murine SGCs (IgG+SGC%; anti-SGC IgG levels) was assessed. The serum from FM subjects and HC was diluted 1:100 in culture media and filtered with a 0.22 μm filter. After washing, the live SGCs were incubated with the serum IgG for three hours. The SGCs were fixed with 4% formaldehyde for ten minutes, followed by washing with 0.1% Triton-X100 in 1X phosphate buffered saline for 5 minutes. The SGCs were incubated overnight with diluted 1:500 rabbit glutamine synthesis IgG (Abcam ab73593) at 4℃. After the SGC were washed, they were incubated once again but now with anti-human IgG (AF594, Thermo Fisher A11014) and anti-rabbit IgG antibody (AF488, Thermo Fisher A11008) diluted 1:300. Finally, the SGCs were washed, counterstained with Hoescht (10 min), and left to dry (10 min) and coverslipped with Prolong gold mounting media. Imaging was done with a Zeiss LSM800 confocal microscope and then analyzed with a custom machine-learning pipeline [(Hunt et al., 2022)](https://paperpile.com/c/BPSce1/uOrIt).

## Mass spectrometry

### Short-chain fatty acids

Two µL of sample was injected on a reversed-phase HPLC column (Accucore C18 100 × 2.1 mm, 2.6 µm, Thermo Scientific). The mobile phases contained 0.1% (v/v) formic acid in water (mobile phase A) and 1:9 isopropanol:MeOH (mobile phase B), respectively. A 14-minute long chromatographic program was applied as follows: 2% B for 0.5 min, 2 - 40% B over 5 min, 40 - 100% B over 3 min, 100% B for 2.5 min, and re-equilibration at 2% B for 2 min. The flow rate was 0.6 mL/min, and the column temperature was 55°C. The HRMS analysis was performed in the full scan mode of *m/z* 100 - 1000 with two selected ion monitoring (SIM) windows: 3.9 - 4.9 min *m/z* 221 - 224 and 4.9 - 6.3 min *m/z* 235 - 238. The spray voltage was 2.4 kV, the capillary temperature and the auxiliary gas heater were set to 320°C and 450°C, the sheath gas flow rate and the auxiliary gas flow rate were set to 55 and 15, respectively, the sweep gas flow rate was 3, and the S-lens RF level was 50.

# Supplementary figures


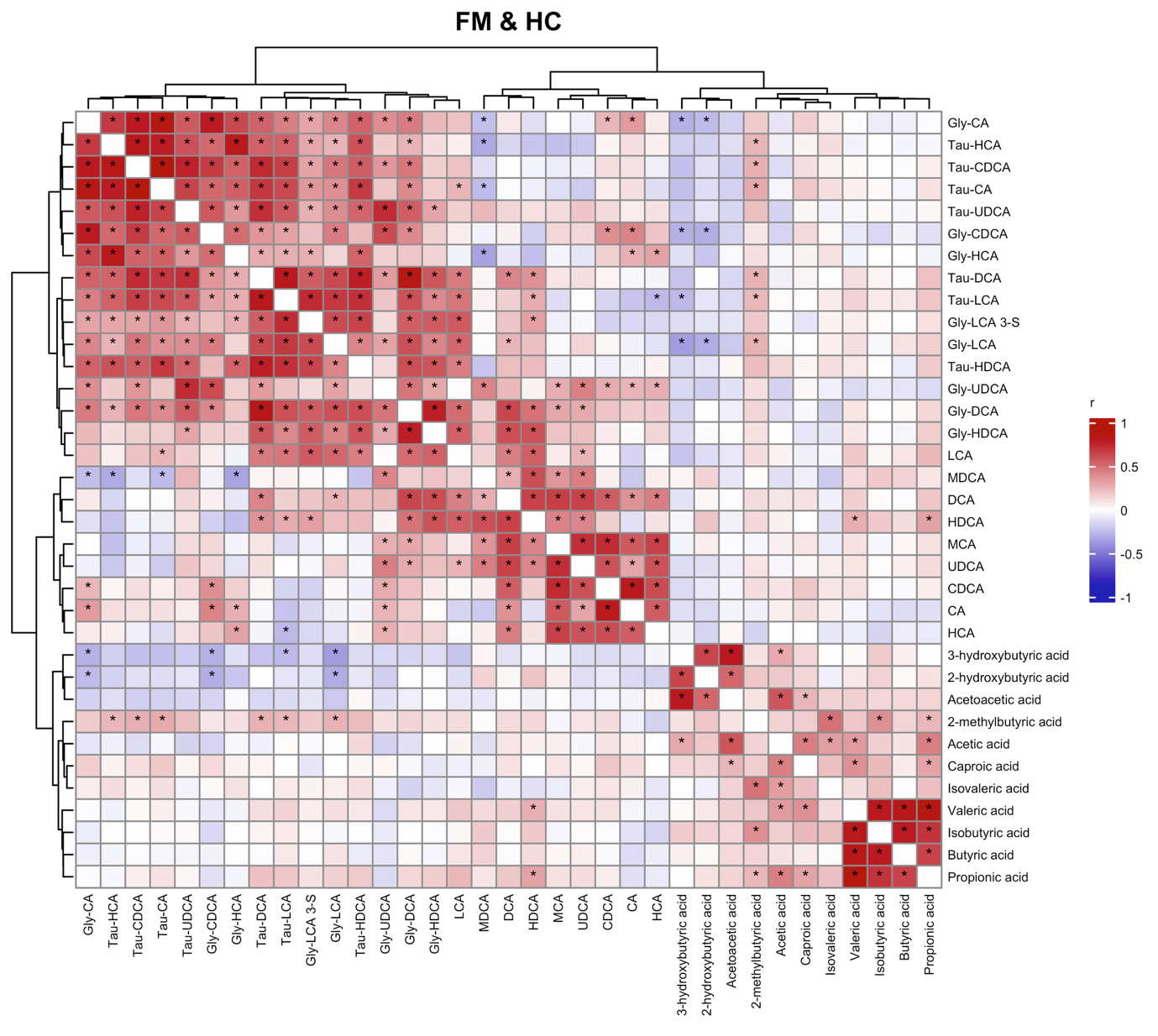


Supplementary Figure S1. Correlating bile acids (BAs) and short-chain fatty acids (SCFAs) show dependencies between each other. The compounds in both fibromyalgia (FM) subjects and healthy controls (HC) were correlated with Spearman’s correlation, and the coefficients (r) were clustered with hierarchical clustering. Abbreviations - *: P < 0.05, CA: cholic acid, CDCA: chenodeoxycholic acid, DCA: deoxycholic acid, Gly: glycine, HCA: hyocholic acid, HDCA: hyodeoxycholic acid, LCA: lithocholic acid, LCA 3-S: lithocholic acid 3-sulfate, MCA: muricholic acid, MDCA: murideoxycholic acid, Tau: taurine, UDCA: ursodeoxycholic acid.


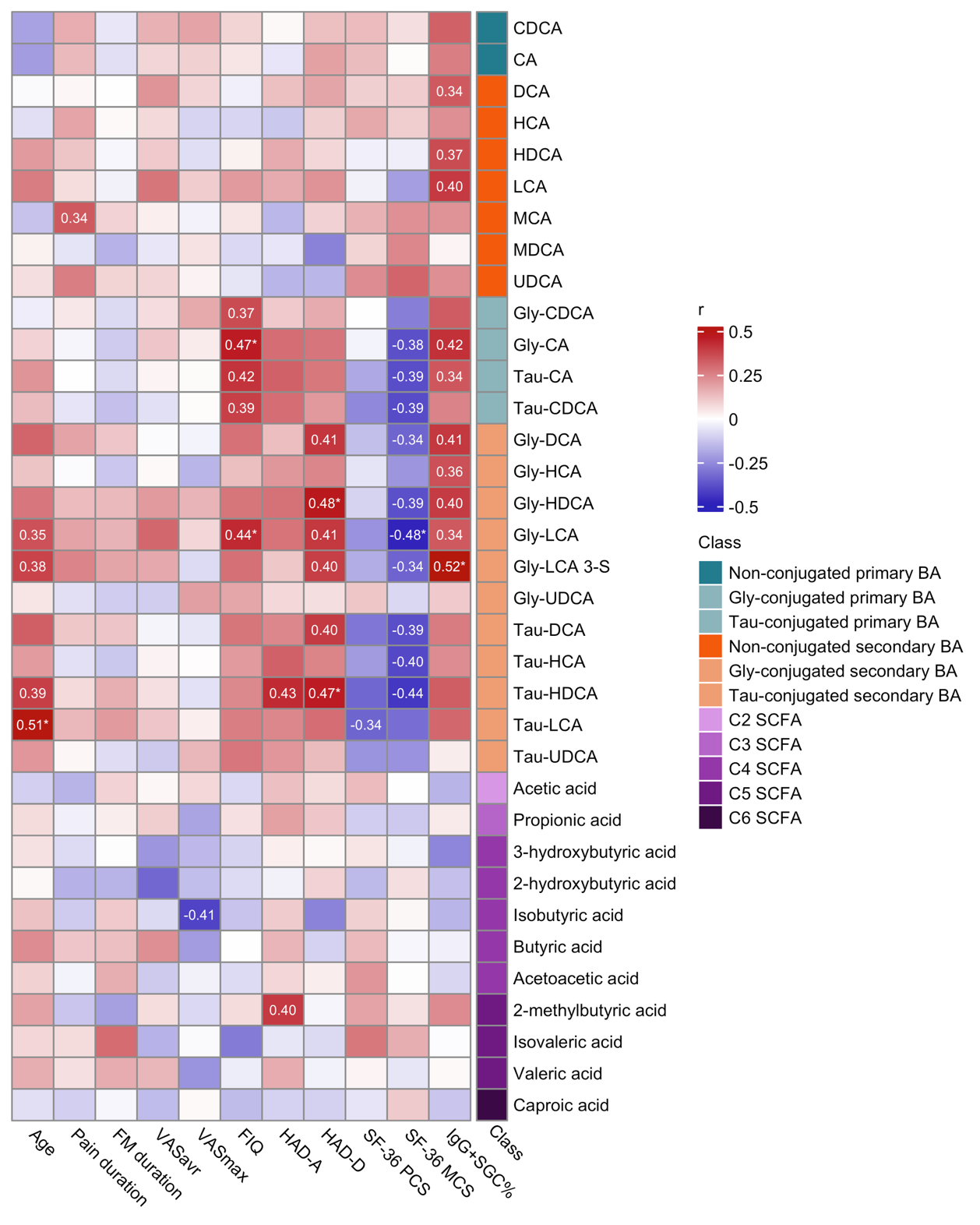


Supplementary figure S2. Correlations between individual bile acids (BAs) and short-chain fatty acids (SCFAs) and clinical measures in fibromyalgia (FM) subjects. Red indicates positive correlations and blue indicates negative correlations. Correlation coefficients are shown for correlations with P < 0.05. An asterisk (*) denotes correlations that remained significant after multiple-testing correction (P≤0.01). Abbreviations - *: P ≤ 0.01, CA: cholic acid, CDCA: chenodeoxycholic acid, DCA: deoxycholic acid, FIQ: Fibromyalgia Impact Questionnaire, Gly: glycine, HCA: hyocholic acid, HDCA: hyodeoxycholic acid, LCA: lithocholic acid, LCA 3-S: lithocholic acid 3-sulfate, MCA: muricholic acid, MDCA: murideoxycholic acid, Tau: taurine, UDCA: ursodeoxycholic acid, VAS: visual analogue scale (pain intensity ratings), VASavr: average weekly pain intensity, VASmax: maximum pain intensity during the past week, SF-36 MCS: mental component score of the short form-36 health survey; SF-36 PCS: physical component score of the short form-36 health survey.

# Supplementary Tables

**Supplementary Table S1.** The authentic reference standards and isotopically marked internal standards were used for the LC-MS analysis. The substances were purchased from Merck (Burlington, MA, United States), Cayman Chemical Company (Ann Arbor, MI, United States), Toronto Research Chemicals (Toronto, ON, Canada), (Larodan, Solna, Sweden), or Avanti polar lipids (Alabaster, AL, USA).

| **Compound** | **Abbreviation** | **Type** | **Manufacturer** |
| --- | --- | --- | --- |
| Chenodeoxycholic acid | CDCA | Reference standard | Merck |
| Cholic acid | CA | Reference standard | Merck |
| Deoxycholic acid | DCA | Reference standard | Merck |
| Hyocholic acid | HCA | Reference standard | Merck |
| Hyodeoxycholic acid | HDCA | Reference standard | Merck |
| Lithocholic acid | LCA | Reference standard | Merck |
| α-muricholic acid | α-MCA | Reference standard | Cayman Chemical Company |
| β-muricholic acid | β-MCA | Reference standard | Cayman Chemical Company |
| ω-muricholic acid | ω-MCA | Reference standard | Cayman Chemical Company |
| Murideoxycholic acid | MDCA | Reference standard | Cayman Chemical Company |
| Ursodeoxycholic acid | UDCA | Reference standard | Merck |
| Gly-chenodeoxycholic acid | Gly-CDCA | Reference standard | Merck |
| Gly-cholic acid | Gly-CA | Reference standard | Merck |
| Gly-cholic acid-d4 | Gly-CA-d4 | Internal standard | Avanti polar lipids |
| Tau-cholic acid | Tau-CA | Reference standard | Merck |
| Tau-cholic acid-d4 | Tau-CA-d4 | Internal standard | Avanti polar lipids |
| Tau-chenodeoxycholic acid | Tau-CDCA | Reference standard | Toronto Research Chemicals |
| Tau-chenodeoxycholic acid-d4 | Tau-CDCA-d4 | Internal standard | Avanti polar lipids |
| Gly-deoxycholic acid | Gly-DCA | Reference standard | Toronto Research Chemicals |
| Gly-hyocholic acid | Gly-HCA | Reference standard | Cayman Chemical Company |
| Gly-hyodeoxycholic acid | Gly-HDCA | Reference standard | Toronto Research Chemicals |
| Gly-lithocholic acid | Gly-LCA | Reference standard | Cayman Chemical Company |
| Gly-lithocholic acid 3-sulfate | Gly-LCA 3-S | Reference standard | Merck |
| Gly-ursodeoxycholic acid | Gly-UDCA | Reference standard | Merck |
| Gly-ursodeoxycholic acid-d4 | Gly-UDCA-d4 | Internal standard | Avanti polar lipids |
| Tau-deoxycholic acid | Tau-DCA | Reference standard | Merck |
| Tau-hyocholic acid | Tau-HCA | Reference standard | Cayman Chemical Company |
| Tau-hyodeoxycholic acid | Tau-HDCA | Reference standard | Toronto Research Chemicals |
| Tau-lithocholic acid | Tau-LCA | Reference standard | Merck |
| Tau-ursodeoxycholic acid | Tau-UDCA | Reference standard | Toronto Research Chemicals |
| Acetic acid |  | Reference standard | Merck |
| Acetic acid-d3 |  | Internal standard | Merck |
| Propionic acid |  | Reference standard | Merck |
| Propionic acid-d5 |  | Internal standard | Larodan |
| 2-hydroxybutyric acid |  | Reference standard | Merck |
| 3-hydroxybutyric acid |  | Reference standard | Merck |
| Acetoacetic acid |  | Reference standard | Merck |
| Butyric acid |  | Reference standard | Merck |
| Butyric acid-d7 |  | Internal standard | Larodan |
| Isobutyric acid |  | Reference standard | Merck |
| 2-methylbutyric acid |  | Reference standard | Merck |
| Isovaleric acid |  | Reference standard | Merck |
| Valeric acid |  | Reference standard | Merck |
| Caproic acid |  | Reference standard | Merck |
| Caproic acid-d11 |  | Internal standard | Larodan |

Supplementary Table S2. Spearman correlations (r) between individual bile acids and short-chain fatty acids (SCFAs) and the frequency of immunoglobulin G binding to satellite glial cells (IgG+SGC%) in fibromyalgia (FM) and healthy controls (HC). Raw p-values (P) were corrected for multiple testing (P_adj_), and P_adj_ < 0.05 was considered significant.

| **Compound** | **FM** | | **HC** | |
| --- | --- | --- | --- | --- |
|  | **r** | **P** | **r** | **P** |
| CDCA | 0.32 | 0.059 | -0.13 | 0.482 |
| CA | 0.26 | 0.132 | -0.15 | 0.399 |
| DCA | 0.34 | 0.048 | 0 | 0.998 |
| HCA | 0.23 | 0.192 | -0.02 | 0.921 |
| HDCA | 0.37 | 0.031 | 0.11 | 0.552 |
| LCA | 0.4 | 0.018 | -0.1 | 0.599 |
| MCA | 0.22 | 0.208 | 0.08 | 0.661 |
| MDCA | 0.02 | 0.909 | 0.32 | 0.075 |
| UDCA | 0.23 | 0.187 | 0.01 | 0.938 |
| Gly-CDCA | 0.33 | 0.051 | -0.27 | 0.136 |
| Gly-CA | 0.42 | 0.011 | -0.27 | 0.135 |
| Tau-CA | 0.34 | 0.045 | -0.31 | 0.088 |
| Tau-CDCA | 0.25 | 0.143 | -0.29 | 0.112 |
| Gly-DCA | 0.41 | 0.015 | -0.17 | 0.366 |
| Gly-HCA | 0.36 | 0.034 | -0.26 | 0.156 |
| Gly-HDCA | 0.4 | 0.017 | -0.06 | 0.729 |
| Gly-LCA | 0.34 | 0.046 | -0.19 | 0.299 |
| Gly-LCA 3-S | 0.52 | 0.001* | -0.21 | 0.249 |
| Gly-UDCA | 0.11 | 0.537 | -0.01 | 0.952 |
| Tau-DCA | 0.27 | 0.123 | -0.23 | 0.212 |
| Tau-HCA | 0.23 | 0.179 | -0.43 | 0.017 |
| Tau-HDCA | 0.33 | 0.061 | -0.19 | 0.354 |
| Tau-LCA | 0.31 | 0.07 | -0.32 | 0.071 |
| Tau-UDCA | 0.04 | 0.844 | -0.21 | 0.256 |
| Acetic acid | -0.16 | 0.346 | -0.08 | 0.652 |
| Propionic acid | 0.04 | 0.818 | -0.09 | 0.635 |
| 3-hydroxybutyric acid | -0.27 | 0.121 | -0.02 | 0.928 |
| 2-hydroxybutyric acid | -0.14 | 0.427 | 0 | 0.979 |
| Isobutyric acid | -0.16 | 0.349 | -0.07 | 0.695 |
| Butyric acid | -0.03 | 0.854 | -0.06 | 0.735 |
| Acetoacetic acid | -0.09 | 0.609 | -0.13 | 0.471 |
| 2-methylbutyric acid | 0.23 | 0.174 | -0.2 | 0.278 |
| Isovaleric acid | -0.01 | 0.966 | -0.08 | 0.667 |
| Valeric acid | 0.01 | 0.964 | -0.07 | 0.719 |
| Caproic acid | -0.13 | 0.465 | -0.22 | 0.236 |

**: Significant after adjustment (P≤0.01), Tau: taurine, Gly: glycine*

Supplementary Table S3. Spearman correlations (r) between summarized bile acid (BA) classes and short-chain fatty acid (SCFA) concentrations and the frequency of immunoglobulin G binding to satellite glial cells (IgG+SGC%) in fibromyalgia (FM) and healthy controls (HC).

| **Class** | **FM** | | **HC** | |
| --- | --- | --- | --- | --- |
|  | **r** | **P** | **r** | **P** |
| Total SCFA | -0.24 | 0.168 | -0.07 | 0.72 |
| Total BA | 0.52 | 0.001* | -0.27 | 0.137 |
| Non-conjugated primary BA | 0.31 | 0.074 | -0.16 | 0.388 |
| Non-conjugated secondary BA | 0.41 | 0.015 | 0.07 | 0.704 |
| Conjugated primary BA | 0.4 | 0.018 | -0.32 | 0.073 |
| Gly-conjugated primary BA | 0.4 | 0.016 | -0.3 | 0.094 |
| Tau-conjugated primary BA | 0.29 | 0.095 | -0.31 | 0.089 |
| Conjugated secondary BA | 0.42 | 0.012 | -0.2 | 0.275 |
| Gly-conjugated secondary BA | 0.43 | 0.009* | -0.16 | 0.388 |
| Tau-conjugated secondary BA | 0.28 | 0.108 | -0.24 | 0.177 |

**: Significant after adjustment (P ≤ 0.01), Tau: taurine, Gly: glycine*
